# Cloning, expression and characterization of l-asparaginase from *Withania somnifera* L. for large scale production

**DOI:** 10.1007/s13205-011-0003-y

**Published:** 2011-04-07

**Authors:** Vishal P. Oza, Pritesh P. Parmar, Darshan H. Patel, R. B. Subramanian

**Affiliations:** Department of Plant Biotechnology, B R D School of Biosciences, Sardar Patel Maidan, Vadtal Road, Satellite Campus, Sardar Patel University, Post Box No. 39, Vallabh Vidyanagar, 388 120 Gujarat India

**Keywords:** l-Asparaginase, cDNA, Cloning, Expression, *Withania somnifera* L.

## Abstract

l-Asparaginase (E.C. 3.5.1.1) is used as a therapeutic agent in the treatment of acute childhood lymphoblastic leukemia. It is found in a variety of organisms such as microbes, plants and mammals. In plants, l-asparaginase enzymes are required to catalyze the release of ammonia from asparagine, which is the main nitrogen-relocation molecule in these organisms. An Indian medicinal plant, *Withania somnifera* was reported as a novel source of l-asparaginase. l-Asparaginase from *W. somnifera* was cloned and overexpressed in *E. coli*. The enzymatic properties of the recombinant enzyme were investigated and the kinetic parameters (*K*_m_, *k*_cat_) for a number of substrates were determined. The kinetic parameters of selected substrates were determined at various pH and the pH- and temperature-dependence profiles were analyzed. WA gene successfully cloned into *E. coli* BL21 (DE3) showed high asparaginase activity with a specific activity of 17.3 IU/mg protein.

## Introduction

The interest in l-asparaginases arose due to their antitumor activity. Unlike normal cells, malignant cells can only slowly synthesize l-asn and are dependent on an exogenous supply (Lee et al. 1989). In contrast, normal cells are protected from Asn-starvation due to their ability to produce this amino acid (Duval et al. 2002). The antineoplastic activity results from depletion of the circulating pools of l-asn by l-asparaginase (Lee et al. 1989). The l-asparaginases of *Erwinia* and *E. coli* have been employed for many years as effective drugs in the treatment of acute lymphoblastic leukemia and leukemia lymphosarcoma (Kristiansen et al. 1970; Kozak et al. 2002; Graham 2003), but their therapeutic response rarely occurs without some evidence of toxicity (Duval et al. 2002). Their main side effects are anaphylaxis, pancreatitis, diabetes, leucopoenia, neurological seizures and coagulation abnormalities that may lead to intracranial thrombosis or hemorrhage (Duval et al. 2002). Because the l-asparaginases from *E. coli* and *Erwinia* possess different immunological specificities, they offer an important alternative therapy if a patient becomes hypersensitive to one of the enzymes (Lee et al. 1989).

In comparison to the bacterial enzymes, the plant enzymes have been studied less thoroughly. In plants, l-asparagine is the major nitrogen storage and transport compound and it may also accumulate under stress conditions (Bruneau et al. 2006). There are two groups of such proteins, called potassium-dependent and potassium-independent asparaginases (Sodek et al. 1980; Sieciechowicz et al. 1988). Both enzyme groups have significant levels of sequence similarity. The plant asparaginase amino acid sequences did not have any significant similarity with microbial asparaginase but was 23% identical and 66% similar to a human glycosylasparaginase (Lough et al. 1992). The crystal structure of plant l-asparaginase showed significant similarity with bacterial as well as threonine aspartase (Karolina et al. 2006).

*Withania somnifera* (L.) Dunal was considered a rasayana herb, which works on a non-specific basis to increase health and longevity. The species name somnifera means “sleep-making” in Latin, indicating to its sedating properties. Extracts of the fruits, leaves and seeds of *W. somnifera* L. were traditionally used in the Ayurvedic system as aphrodisiacs, diuretics and for treating memory loss. *W. somnifera* L. was reported as a potential source for l-asparaginase (Oza et al. 2009) and the purified enzyme was shown to have anti-tumor activity on cell cultures (Oza et al. 2010).

The toxicity is partially attributable to the glutaminase activity of these enzymes (Howard and Carpenter 1972). l-asparaginases with high specificity for l-asparagine and negligible activity against l-glutamine are reported to be less troublesome during the course of anti-cancer therapy (Hawkins et al. 2004). The interest in l-asparaginase from *W. somnifera* L. arose from the fact that it has less toxicity compared to bacterial l-asparaginase (Oza et al. 2010). Despite the therapeutic potential of WA, this enzyme is less characterized, compared to other l-asparaginases. Detailed studies of protein and large scale production of the therapeutic protein through recombinant enzyme have not been reported so far from *W. somnifera* L. Therefore, in this study expression, purification and characterization of recombinant l-asparaginase from *W. somnifera* is reported for the first time.

## Materials and methods

### Materials

All degenerate oligonucleotides and l-asparaginase-specific primers used in this study were synthesized by MWG (India). Oligo (dT)-anchor primer and all other chemicals such as M-MuLV reverse transcriptase, DTT, RNase inhibitor and many more for amplification of cDNA were obtained from Bangalore Genei (India). *Taq* DNA polymerase and MgCl_2_ were from Sigma-Aldrich Co (USA). Restriction enzyme *Hin*dIII and *Xho*lI was obtained from NEB (UK). Qiagen Plasmid Maxi Kit and transformation chemicals were from Qiagen (Germany). l-Asparagine was obtained from Sigma-Aldrich Co.

*Withania somnifera* (L.) Dunal was collected from the Botanical garden, Department of Biosciences, Sardar Patel University, Vallabh Vidyanagar, Gujarat (India). A voucher specimen of the plant was submitted to the department herbarium. Identification of the plant was confirmed through comparison with a herbarium specimen number 10337.

### RNA isolation and cDNA synthesis

One gram of immature, 10-day-old fruits was washed thoroughly with tap water followed by sterile distilled water to remove extraneous material and homogenized in liquid nitrogen using a homogenizer. Extraction of total RNA was carried out using TRI reagent (Sigma). Sequences homologous to WA were sought in the NCBI using BLAST. A cDNA sequence of l-asparaginase from *A.thaliana* (GenBank accession no: Z34884) was used for cDNA synthesis. Reverse transcriptase PCR was used to amplify the full-length cDNA. 1 U of DNase was added into total RNA sample (5 μg), mixed properly and incubated for 15 min at room temperature. DNase was inactivated by incubation at 75 °C for 10 min and it was carried out before RT-PCR. The cDNA synthesis was carried out in a total volume of 20 μl, containing 100 ng of total RNA, 100 pmol of random hexamer, DEPC treated water, RNase inhibitor, DTT, RT Buffer, dNTP mix and M-MuLV reverse transcriptase. cDNA was amplified through PCR using the heterologous primers synthesized to the 5′-region of the cDNA (5′-ATG GGC GGC TGG AGC ATT GC-3′) and to the 3′-end of the cDNA (5′-CTT TCA GGC TCA GGC CTT TA-3′), 100 pmol of both primer, 100 ng cDNA, 0.2 mM of each dNTP, 5 μl 10× *Taq* buffer and 2 units of *Taq* DNA polymerase (Bangalore Genei). The PCR procedure comprised 35 cycles of 1.0 min at 95 °C, 1.0 min at 54 °C and 1.0 min at 72 °C. A final extension time at 72 °C for 5 min was performed after the 35 cycles. The resulting PCR amplicon was sequenced in multicolumn sequencer (ABI, USA). The sequence was validated by BLASTn and submitted to NCBI.

### Cloning and overexpression of WsA

For sticky end ligation, the primers were modified in such a manner that they included the RE sites at both end of the PCR amplicon. The following primers were used WSF: 5′-GGC TTC TTA CTC GAG AAT GGG CGG CTG GAG CAT TGC-3′ (*Xho*lI site is underlined) and the reverse primer WSR: 5′-GGC TTC TTA AAG CTT CTT TCA GGC TCA GGC CTT TA-3′ (*Hin*dIII site is underlined). The resulting PCR amplicon which was obtained through RTPCR was re-amplified with the modified primers with the same condition as above. The modified amplified product and plasmid expression vector pRSET A (Invitrogen USA) were digested with *Hin*dIII and *Xho*lI (NEB, USA). The digested PCR product and plasmid vector were run on agarose electrophoresis and purified from the gel. The purified product was ligated into pRSET A using T4 ligase enzyme. The resulting expression construct pRSET A was used to transform competent DH5α *E. coli* cells. The transformed *E. coli* cells, harboring plasmid pRSET A, were grown at 37 °C in 100 ml LB medium containing 100 μg/ml ampicillin. The plasmid was isolated from DH5α *E. coli* and used to transform competent BL21 (DE3) *E. coli*. The cells were grown into LB agar plate containing ampicillin and X-gal. The recombinant clones were identified by blue/white selection and grown at 37 °C in 500 ml LB medium containing 100 μg/ml ampicillin. Synthesis of WsA was induced by the addition of 1 mM IPTG when the absorbance at 600 nm was 0.6–0.8 (Sambrook et al. 1989).

### Purification of WsA

Five hours after induction by IPTG, cells were harvested by centrifugation at 10,000*g* and 4 °C for 20 min. Cells obtained from culture (about 2 g of wet weight) were resuspended in 5 ml potassium phosphate buffer (10 mM, pH 6.5), sonicated and centrifuged at 14,000*g* for 30 min at 4 °C. The supernatant was collected and used for purification. The GeNei™ His-Tag fusion protein purification kit containing Nickel CL-Agarose column (2.5 × 10.0 cm) was used for the purification of WsA. The fractions collected (1 ml) were assayed for asparaginase activity by Nessler’s reagent method (Wriston and Yellin 1973) and protein (*A*_280_) estimation (Lowry et al. 1951). The purified enzyme was dialyzed to remove salt.

### Kinetic analysis

Steady-state kinetic measurements were performed in 10 mM potassium phosphate buffer pH 6.5 at 37 °C, by varying the concentration of the substrate l-asn. The kinetic parameters *k*_cat_, *K*_m_ and *V*_max_ were calculated by non-linear regression analysis of experimental steady-state data. Kinetic data *k*_cat_ and *K*_m_ were calculated using the GraFit program (Erithacus Software Ltd.).

### pH and temperature effect on activity

The pH profile of WsA was studied using the following buffers, all at 0.05 M final concentration at 37 °C with different buffers: 0.01 M sodium acetate (pH 4.0–5.5), 0.01 M Na_2_HPo_4_/NaH_2_Po_4_ (pH 6.0–7.0) and 0.01 M sodium borate buffer (pH 7.5–9.0), in a range of 4.0–9.0 and data were analyzed through statistical software. The dependence of the reaction rate on temperature was evaluated by measuring enzyme activity for l-asn, at different temperature values under the same conditions reported above. The data were analyzed by the SPSS software.

## Results

### cDNA synthesis by reverse transcription

The total RNA yield obtained through TRI method from *W. somnifera* L. was of good quality and suitable for further use. The DNase-treated RNA was used as a template for cDNA synthesis. The cDNA for l-asparaginase obtained was amplified by PCR. The PCR product obtained was a single band of around 850 bp in agarose gel electrophoresis. The PCR amplicon was sequenced using both forward and reverse primers in a multicolumn sequencer (ABI). The sequence was validated with BLAST and submitted to gene bank (FJ645259).

### Cloning and overexpression of WsA

To select the required expression product, prSET-A vector was chosen. The amplified product and prSET-A were digested with *Hin*dIII and *Xho*lI, respectively, and the digested PCR amplicon was cloned into the T7 expression vector pRSET A. The fusion product was named pr/WsA. The pr/WsA plasmid was used to initially transform into *E. coli* DH5α. The plasmid from DH5α *E. coli* cells was purified and used to transform expression host *E. coli* BL21 (DE3). Cell-free extract of the *E. coli* BL21 (DE3) showed high asparaginase activity with a specific activity of 17.3 IU/mg protein.

### Purification of WsA

**Fig. 1:**
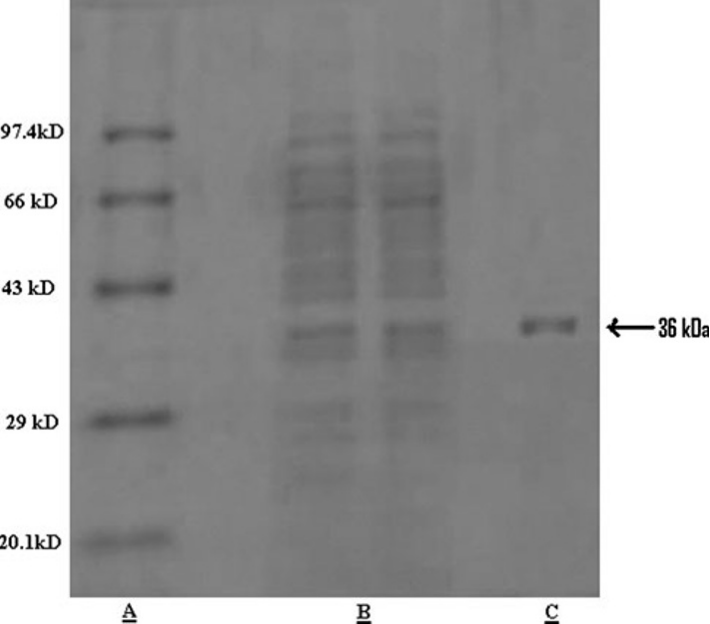
SDS-polyacrylamide gel electrophoresis of l-asparaginase preparations. Protein bands were stained with colloidal stain. *Lane A* Molecular weight markers. *Lane B* WsA crude preparation after induction with 1 mM IPTG. *Lane C* Purified WsA sample

WsA was purified in a one-step procedure (Table 1). In the purification scheme, a His-Tag fusion protein purification Nickel CL-Agarose column was employed. Non-pre-treated enzyme extract was applied directly to the Nickel CL-Agarose column equilibrated with 10 mM potassium phosphate buffer pH 7.5. The enzyme was adsorbed at pH 6.5 and eluted specifically by breaking the His-Tag. The purity of the final WsA preparation was evaluated by SDS-PAGE, which showed the presence of a single polypeptide chain (Fig. 1).

**Table 1:** Purification of recombinant l-asparaginase from *E. coli*

Sample	Volume (ml)	Protein (mg)	Activity (IU)	Specific activity (IU/mg)	Fold purification	Recovery (%)
Cell-free extract	5.0	12.3	212.0	17.3	1.0	100
Nickel CL-agarose	1.0	1.7	95.0	55.8	3.22	44.8

One international unit (IU) of l-asparaginase is that amount of enzyme which liberates 1 μmol of ammonia in 1 min at 37 °C

### Kinetic analysis

The kinetic properties of the WsA were investigated. The *k*_cat_ and *K*_*m*_ parameters for substrate were determined by steady-state kinetic analysis (Table 2). The *K*_m_ values for the recombinant l-asparaginase with l-asparagine and l-glutamine were determined as 0.075 and 4.5 mM, respectively. The catalytic constant *k*_cat_ of WsA is significantly higher than that of the recombinant l-asparaginase from *E. coli* (*K*_m_; 0.085 and 0.058 mM and *k*_cat_; −31.4 and 23.8 × 10^3 ^s^−1^) (Kotzia and Labrou 2005, 2007). The glutaminase activity was responsible for side effects during treatment (Hawkins et al. 2004). The purified enzyme showed very low glutaminase activity, which is about 2% of that of l-asparaginase activity. The activity was significantly lower than that exhibited by the *E. coli* and *Erwinia chrysanthemi* enzymes (Howard and Carpenter 1972).

**Table 2:** Kinetic parameters of WsA and WA

Enzyme	Substrate	*K*_m_ (mM)	*k*_cat_ (s^−1^)	*k*_cat_/*K*_m_ (M^−1 ^s^−1^) (×10^3^)
WA	l-asn	0.06 ± 0.02	17.8 ± 0.1 (×10^3^)	296.6
	l-glu	5.4 ± 0.4	191 ± 3.0	35.4
WsA	l-asn	0.07 ± 0.02	28.6 ± 0.1 (×10^3^)	408.6
	l-glu	4.2 ± 0.3	343 ± 5.0	81.3

Steady-state kinetic measurements were performed at 37 °C. All initial velocities were determined in triplicate. The kinetic parameters *k*_cat_ and *K*_m_ were calculated using the GraFit program (Erithacus Software Ltd.)

### Effect of temperature and pH on enzyme activity

**Fig. 2:**
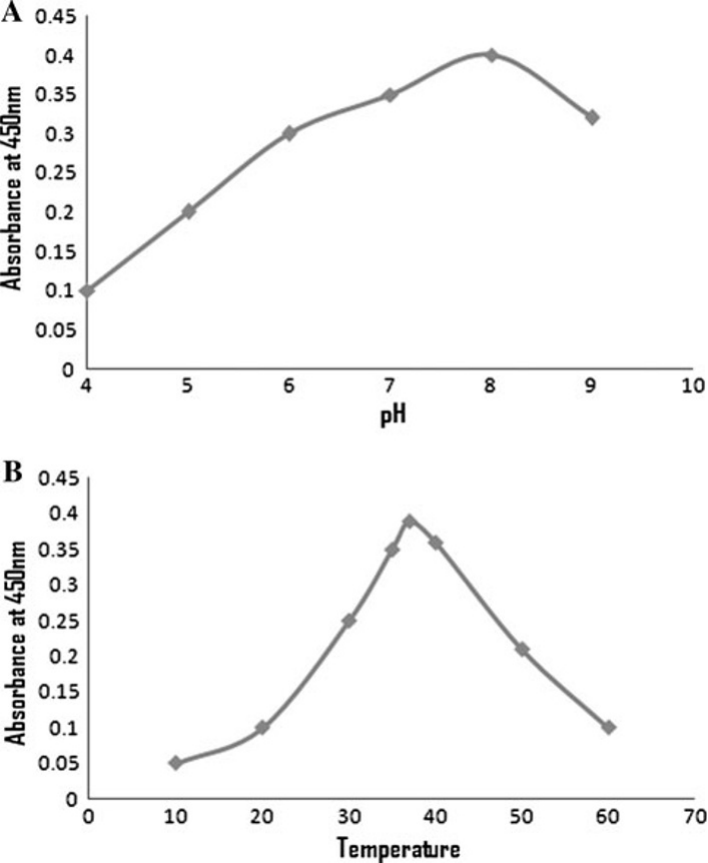
**a** pH-dependence of kinetic parameters of WsA at 37 °C. **b** Effect of temperature on WsA activity at pH 8.0

The optimum pH for WsA enzyme was found to be 8.0 (Fig. 2a) similar to that of *E. coli* and the wild-type enzyme which also had pH optima around 8.5 (Oza et al. 2009). The temperature optimum (37 °C) of the enzyme (Fig. 2b) is also similar to all other reported bacterial sources which are already being used in the treatment of leukemia.

## Discussion

The role of l-asparaginase has been studied for the past 30 years. It plays a unique role in the treatment of acute lymphoblastic leukemia. The production of plant l-asparaginase in large quantities was quite difficult compared to microorganisms. Currently, l-asparaginase for the treatment of ALL is produced from microorganisms. A major drawback of using microorganisms as a source of this drug is a number of adverse effects of the enzyme. As a viable alternative to bacterial l-asp we selected a plant gene as a source to overcome the limitations of the bacterial enzyme. It is evident from the results that the l-asparaginase gene of WA has successfully been cloned and expressed at a high level in *E. coli*. The purified enzyme showed a high level of activity at 95 IU/ml. l-Asparaginase production by *E. coli* and *E. chrysanthemi* has been studied previously. However, research on molecular cloning and gene expression of l-asparaginase from plants has scarcely been reported. Gilbert et al. (1986) and Liu et al. (1996) have cloned and expressed *E. coli* and *E. chrysanthemi*l-asparaginase gene into *E. coli*. The enzyme activities of these recombinant strains were 49 and 106 IU/ml, respectively. In the present study, *lac* and *tac* promoters were used along with IPTG, for the induction of the enzyme. The recombinant strain produced up to eightfold higher activity as compared to the wild-type *E. coli*; hence, the recombinant strain is suitable for the production of l-asparaginase. The purification of l-asparaginase could be achieved relatively easily due to a His-Tag in the recombinant protein. The protein was purified with the help of affinity chromatography.

A comparison of the enzyme characters of recombinant *W. somnifera* and that of *E. coli* and *E. carotovora* indicate that the WsA enzyme is superior in many parameters. While the optimum temperature and pH for the enzyme activity were similar, the l-glutaminase activity was much lower. A major reason for the undesirable side effects of the bacterial enzyme is their glutaminase activity (Howard and Carpenter 1972). Moreover, the enzyme turnover rate of the WsA enzyme was much higher than the bacterial enzyme. The constant *k*_cat_ of WsA is higher than that of l-asparaginase from *E. coli* (Kotzia and Labrou 2005, 2007). The purified enzyme exhibited very low l-glutaminase activity, which is about 2% of that of l-asparaginase activity which is significantly lower than that exhibited by the *E. coli* and *E. chrysanthemi* enzymes (Narta et al. 2007). The *K*_m_ value of WsA indicates that the enzyme efficiency is similar to the wild-type enzyme (Oza et al. 2009). The optimum pH and temperature of WsA were similar to other reported sources such as *E. coli* and wild-type *W. somnifera* L. (Gilbert et al. 1986; Oza et al. 2009).

In conclusion, in the present study, a new l-asparaginase from *W. somnifera* L. has been successfully cloned, expressed and characterized. The results of the present work form the basis for a rational and combinatorial design of new engineered forms of WsA with improved specificity and enhanced catalytic efficiency toward l-asn for future therapeutic use.

## Abbreviations

WA: *Withania somnifera*l-asparaginase
WsA: Recombinant l-asparaginase
Tas1: Threonine aspartase (taspase1)
PDB: Protein Data Bank
LB: Luria broth
